# A Review on the Current Applications of Artificial Intelligence in the Operating Room

**DOI:** 10.1177/1553350621996961

**Published:** 2021-02-24

**Authors:** David C. Birkhoff, Anne Sophie H.M. van Dalen, Marlies P. Schijven

**Affiliations:** 1 Department of Surgery, 26066 Amsterdam UMC, University of Amsterdam, The Netherlands; 2 Department of Surgery, 26066 Amsterdam Gastroenterology and Metabolism, University of Amsterdam, The Netherlands; 3 Li Ka Shing Knowledge Institute, St Michaels Hospital, Toronto, Canada

**Keywords:** artificial intelligence, black box, machine learning, surgery, operating room, innovation, deep learning, neural networks, computer vision

## Abstract

*Background*. Artificial intelligence (AI) is an era upcoming in medicine and, more recently, in the operating room (OR). Existing literature elaborates mainly on the future possibilities and expectations for AI in surgery. The aim of this study is to systematically provide an overview of the current actual AI applications used to support processes inside the OR. *Methods*. PubMed, Embase, Cochrane Library, and IEEE Xplore were searched using inclusion criteria for relevant articles up to August 25th, 2020. No study types were excluded beforehand. Articles describing current AI applications for surgical purposes inside the OR were reviewed. *Results*. Nine studies were included. An overview of the researched and described applications of AI in the OR is provided, including procedure duration prediction, gesture recognition, intraoperative cancer detection, intraoperative video analysis, workflow recognition, an endoscopic guidance system, knot-tying, and automatic registration and tracking of the bone in orthopedic surgery. These technologies are compared to their, often non-AI, baseline alternatives. *Conclusions*. Currently described applications of AI in the OR are limited to date. They may, however, have a promising future in improving surgical precision, reduce manpower, support intraoperative decision-making, and increase surgical safety. Nonetheless, the application and implementation of AI inside the OR still has several challenges to overcome. Clear regulatory, organizational, and clinical conditions are imperative for AI to redeem its promise. Future research on use of AI in the OR should therefore focus on clinical validation of AI applications, the legal and ethical considerations, and on evaluation of implementation trajectory.

## Introduction

The last few years have seen a tremendous growth in the use of sensors, video, and digital devices in the operating room (OR).^1–3^ These applications generate large amounts of data in various formats, often referred to as “big data.”^4^ Big data sets are complex and may be analyzed computationally to reveal patterns, trends, and associations, especially relating to human behavior and interactions. Big data has the potential to become progressively useful in both guiding surgical care and optimizing clinical patient outcomes, if handled well.^5-8^ A limitation often overseen in analyzing big data is that traditional data processing techniques are not able to handle these vast amounts of complex data.^9^ The solution may lie in a research area that became popularly known as “artificial intelligence (AI).” The term AI is often used to describe the study of algorithms that enables machines to reason and perform cognitive functions such as learning, problem-solving, and decision-making.^10,11^ Recently, AI has made its introduction into medicine and, even more recently, into the OR.^2^ This is of interest as these high-risk environments are considered to be one of the most error-prone areas in the hospital, where outcome is highly dependent on use of modern technology generating multisource data.^12,13^ As such, if properly used, AI may have great impact on surgical workflow and outcome. It may provide context-aware perioperative decision support, predict patterns in patient parameters, monitor progress, and develop new in situ training tools.^14–17^ These are just a few examples. To date, AI applications are painting and predicting a promising future surgical landscape. Yet, as is often the case with new innovations, AI may become lost in its promise when it is unclear what the actual baseline and best use case is.^18–20^

The current medical literature fixates predominantly on the future possibilities of AI in surgery, or more specifically, inside the OR. However, it is important to know the current situation—where does AI in the OR stand?—in order to validly decide on areas worthy of further exploration. The aim of this study is to systematically provide an overview of the current AI applications in surgery, used to support various processes inside the OR.

## Methods

### Literature Search

A systematic literary search was performed up to August 25th, 2020 using the following online databases: PubMed, Embase, Cochrane Library, and IEEE Xplore. The terms AI, OR, and surgery, including synonyms or equivalent terms, were used in certain combinations to obtain the relevant literature. The full search strategy can be found in Supplemental Appendix A.

Article screening was done independently by 2 reviewers (DCB and AvD). The inclusion criteria were as follows: (1) AI, (2) in surgery, and (3) in the OR. The exclusion criteria, next to duplicates and articles older than 10 years, were the following: (1) articles published in any language other than Dutch or English, (2) articles containing future applications of AI only, (3) AI used outside the OR, and (4) no full-text availability. Any study design may benefit the study, so no specific study designs were excluded beforehand. Disagreement between the two reviewers in study selection was resolved by healthy discussions concluding in consensus.

The studies that were included after full-text screening were critically appraised, with the use of an Evidence-Based Medicine Critical Appraisal Checklist (see Supplemental Appendix B*).*

### Data Extraction

The included articles were extracted of data on study design, publication year, country of origin, and the specific researched applications of AI. The outcomes of these studies were analyzed and described and, if possible, defined in numbers. A clear overview of the different studies, their applications of AI and their specifically used subfield of AI, and their data type/source is provided. AI, while not easily defined, is a machine’s capability to mimic intelligent human behavior.^21^ AI is a broad field to be distinguished by multiple subfields. In order to better understand the analyses and outcomes of the studies, it was decided to explain some of the different subfields in AI beforehand. The subfields that are of importance to this systematic review are explained and elaborated on in Table 1.

**Table 1.** Definitions of major subfields in artificial intelligence.

Subfield	Definition
Machine learning (ML)	Gives computers via algorithms the ability to modify its processing when exposed to more information, without being specifically programmed to do so.^57,58^ In this way, computers are capable of “learning from experience.”^21,58,59^ ML is considered to be promising in pattern recognition in large cohorts of data by using more complex techniques than traditional statistical analysis does.^60,61^
Artificial neural networks (ANNs)	Are tools used in ML. In function, they are imitating the human brain by connecting and finding interrelated complex relationships and patterns between data.^2^ Basically, ANNs are composed of many computational units (neurons) that receive inputs, perform calculations, and direct output to the next computational unit. In other words, the input is being processed as signals through layers of algorithms that create certain patterns as final output; these patterns are interpreted and used in decision-making.^62^ ANNs are commonly composed of simple 1- or 2-layered neural networks.
Deep learning	Deep learning networks consist of many layers and are able to recognize and learn more subtle and complex patterns.^63^ Deep learning networks may take one or more datasets into account, which are evaluated multiple times in many different layers, until reaching the desired output.^21^
Convolutional neural networks (CNNs)	A convolutional neural network (CNN) is a class of ANNs that specializes in processing data in visualized imagery. In deep learning, “convolution” is a specialized kind of linear operation used in analyzing images, and in CNNs, the ANN employs this mathematical operation in at least one of its layers, hence the name convolutional.^64^
Computer vision (CV)	Focuses on how computers can gain high-level understanding of digital images and videos such as object and scene recognition, comparable to the human visual system.^25,65,66^ The processed data may consist of video sequences, views from multiple cameras, or multidimensional data from a medical scanning device.^67,68^

Abbreviations: ML = machine learning; ANN = artificial neural networks; CV = computer vision; CNN = convolutional neural network

## Results

### Search Results and Study Selection

**Figure 1.**
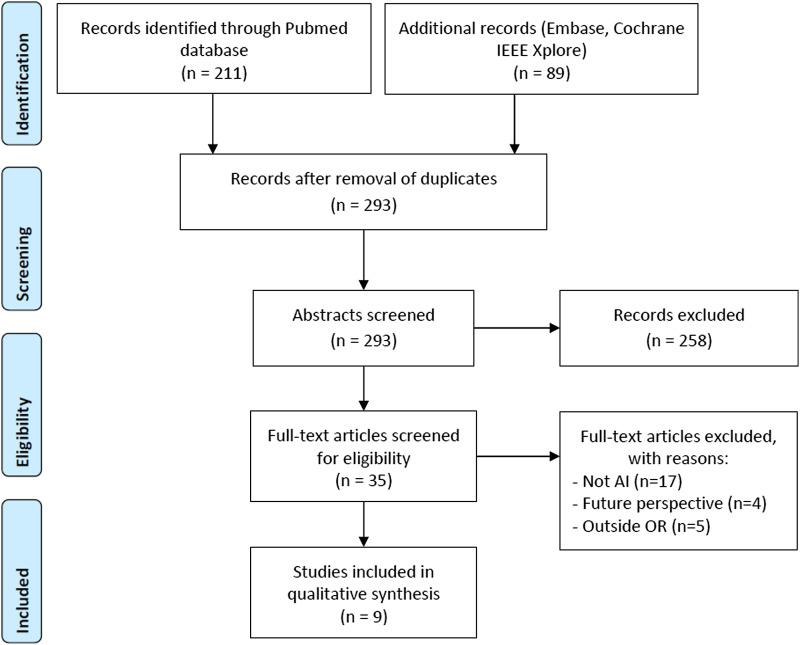
Flowchart of literature search.

The literature search yielded 193 articles from PubMed database, 50 articles from Embase database, 5 articles from the Cochrane Library, and 27 articles from IEEE Xplore database. Finally, 9 articles were included. The flowchart with a more detailed description of the selection procedure may be viewed in Figure 1. The nine included studies are the following: Bodenstedt et al.,^22^ Cho et al.,^23^ Devi et al.,^24^ Hashimoto et al.,^25^ Jermyn et al.,^26^ Kassahun et al.,^27^ Padoy,^17^ Zhao et al.,^28^ and Liu et al.^29^

### Critical Appraisal

Only the 2 included review studies by Padoy^17^ and Kassahun et al.,^27^ were critically appraised. As a consequence of inhomogeneity in study design, the additional seven included studies did not contain a sufficient amount of checklist characteristics and were therefore not suitable for critical appraisal. Although both review studies scored negatively on many criteria, indicating that the quality of the studies should be considered relatively low, these studies were not of a regular review design either and were therefore difficult to classify.

### Applications of AI

The included articles respectively researched one or multiple applications of AI in surgery. Table 2 shows an overview of the different studies, their researched application(s), and the specific AI subfield(s) the application is based on. Additionally, Table 2 specifies the data type/source that was used by the AI application.

**Table 2.** Overview of included studies with specific AI application(s).

Application(s)	Study	AI Subfield(s) ^a^	Data Type/Source
Procedure duration prediction	Bodenstedt et al.^22^	CNN, deep learning, and ML	Video stream
	Zhao et al.^28^	ANN and ML	Case characteristics^b^
	Devi et al.^24^	ANN and ML	Surgical environment^c^
Gesture recognition	Cho et al.^23^	CNN and ML	Depth video stream and radiological images
Intraoperative cancer detection	Jermyn et al.^26^	ANN and ML	Spectral light
Intraoperative video analysis	Hashimoto et al.^25^	ANN, CV, and ML	Video stream
Workflow recognition	Padoy^17^	CNN, CV, RNN, deep learning, and ML	Video stream and images
Endoscopic guidance system and knot-tying	Kassahun et al.^27^	RNN and ML	Video stream
Automatic registration and tracking of the bone in orthopedic surgery	Liu et al.^29^	ANN, deep learning, and ML	Depth camera images

^a^ Abbreviations: CNN = convolutional neural network, ML = machine learning, ANN = artificial neural network, CV = computer vision, RNN = recurrent neural network.

^b^ Scheduled duration, age, gender, and comorbidities of the patient, tumor location, month of year, time of day, day of the week, etc.

^c^ Experience of surgeon in years, experience of anesthetist in years, staff experience in years, type of anesthesia, etc. The actual set of environment variables depends on the type of surgery.

### Procedure Duration Prediction

Due to the high density and non-singularity of information in a video stream, extracting its data for evaluation purposes is a challenging process. In comparison to the video stream, data from surgical instruments provide information that is easier to quantify. Whether or not such data provide sufficient information to make presumptive predictions on surgery duration is uncertain to date. Bodenstedt et al.^22^ proposed and compared methods, based on CNNs to predict procedure duration based on data from surgical devices or video streams. The input was acquired from 80 recorded laparoscopic interventions of which the necessary data were available. Overall, the combined method (both video and surgical device data) performed best with an average error of 37% and an average halftime error of approximately 28%. This is an improvement to the baseline method with an average error and average halftime error of both 124%.^22^

Zhao et al. sought to accurately predict procedure duration of robot-assisted surgery cases using multiple machine learning (ML) models, using case characteristics (scheduled duration, age, gender, and comorbidities of the patient, tumor location, month of year, time of day, day of the week etc.) as data input. They compared the ML models to the baseline model, which is the time scheduled for the procedure determined by former case duration averages and changes by the surgeon. The following ML models were used: (1) multivariable linear regression, (2) ridge regression, (3) lasso regression, (4) random forest, (5) boosted regression tree, and (6) ANNs. The average root-mean-squared error (RMSE), a measure for the imperfection of the fit of the estimator to the data, was lower for all the ML models than the baseline model. The average RMSE was lowest with the boosted regression tree (80.2 minutes, 95% confidence interval 74.0–86.4), which was significantly lower than the baseline model (100.4 minutes, 95% Confidence interval 90.5–110.3). The use of a boosted regression tree, apredictive modeling approach used in ML, increased the amount of correctly booked procedures from 148 to 219 (34.9% to 51.7%, *P* <.001).^28^

Devi et al. researched several techniques to estimate procedure duration in an ophthalmology department by taking the surgical environment into account (experience of surgeon in years, experience of anesthetists in years, type of anesthesia, etc.). Three techniques were researched, namely, adaptive neuro-fuzzy inference systems (ANFISs), multiple linear regression analysis (MLRA), and ANNs. However, ANFIS is a fusion between the adaptive learning capability of ANNs and the intuitive logic of human reasoning, formulated as a feed-forward neural network. The results of procedure duration prediction were compared between the three techniques, and the ANFIS model came out to be performing better than the other 2 as portrayed in Table 3.^24^

**Table 3.** Comparison of techniques to estimate procedure duration.^24^

Type of Surgery	Root-Mean-Squared Error (RMSE)		
	ANFIS	ANN	Regression
Corneal transplant	.1557	.1895	.2755
Cataract	.0697	.1427	.1768
Oculoplastic	.1431	.1668	.2123

Abbreviations: ANN = artificial neural networks; ANFIS = adaptive neuro-fuzzy inference systems.

### Gesture Recognition

To decrease the risk of contamination during surgical procedures, Cho et al.^23^ researched a noncontact interface based on ML models in order to enhance the accuracy of gesture recognition. Support vector machines (SVMs) and naive Bayes classifiers, ML models with associated algorithms used for classification, were used in the study.^30^ Cho et al. used 30 features, including hand and finger data, as input for these ML models to predict and train 5 types of gestures. The overall accuracy of the 5 gestures was 99.58% ± .06 and 98.74% ± 3.64, respectively, for SVM and naive Bayes classifiers. Self-training methods of SVMs and naive Bayes classifiers improved accuracies by about 5–10%.^23^

### Intraoperative Cancer Detection

During brain tumor removal it is important yet very difficult to detect and remove all cancer cells. As a consequence, when not completely removed, the patient is at risk for recurrence of cancer. With certain types of brain cancer in vivo, Raman spectroscopy can detect these invasive cancer cells. A downside to this technique is the fact that the Raman signal is weakened by spectral artifacts generated by the regular lights in the OR. Jermyn et al. found that ANNs are able to improve the detection of invasive brain cancer cells by overcoming the negative impact of spectral artifacts. Despite the inclusion of light artifacts, ANNs keep the detection of invasive cancer cells at almost the same level, improving sensitivity by 19% and specificity by 7% compared to the standard technique.^26^

### Intraoperative Video Analysis

Video data of laparoscopic procedures are used for both education and quality improvement purposes. In order to decrease the required time for analysis and review of video data, Hashimoto et al. investigated the possibility of automatic video segmentation using CV and ML techniques. Their research demonstrated that CV and ML techniques were able to differentiate between specific steps of laparoscopic surgery procedures with an accuracy of 82% ± 4%.^25^

### Workflow Recognition

The long-term vision of Padoy^17^ is to develop a surgical control tower (SCT) that, using AI, can monitor and support many processes, providing overall awareness of what is happening in the OR. Key for such an SCT is the requirement of an AI system that can recognize the surgical workflow and is aware of the surgical context. Workflow is often described as the sequence of tasks, interactions, or other processes through which a piece of work passes from initiation to completion.^31^ In their review, Padoy^17^ researched several recent ML and deep learning applications that can add to the workflow recognition system. These applications include phase recognition, tool detection and localization, and human detection and pose estimation and are described below.^17^

### Phase Recognition

Phase recognition, the task of instantly determining the current phase of surgery at any time *t* from video data, was researched both in laparoscopic videos and external videos. In laparoscopic videos, a study showed that the combination of a CNN and a recurrent neural network (RNN) was able to recognize the different phases automatically and in real time, with an accuracy of 86%. In a study using external videos, a combination of a CNN and hidden Markov models (HMMs), a popular application for ordinal or temporal data within AI, recognized different phases in the surgical procedure with an accuracy of 90%.^17^

### Tool Detection and Localization

Tool detection and localization adds to the precision of phase recognition. By recognizing more subtle and detailed activities, tool detection and localization may be informative for predicting operative steps and length of operation. Deep learning techniques were used to research tool detection and localization in laparoscopic images and videos. Using a CNN, results show a mean average precision of 87% in tool detection and 88% in tool localization.^17^

### Human Detection and Pose Estimation

Since the people are the main actors in the OR, detecting their position and estimating their poses by localizing their body parts can provide useful information for optimizing workflow. With the use of external videos, the ability to estimate the specific body poses of the people in the OR was investigated. The mean per joint position error (MPJPE) was used as a quantitative measure for 2D and 3D body part localization. Deep learning approaches yielded the best results in both 2D and 3D pose estimation with an average MPJPE of 17 and 5 cm, respectively.^17^

### Endoscopic Guidance System

Weede et al. described an autonomous endoscopic guidance system based on ML. The system is capable of collecting and processing data on the movements of surgical instruments in recorded videos of surgical procedures. Subsequently, with the use of trajectory clustering, maximum likelihood classification, and HMMs, the system uses this information to predict trajectories that are used to guide the endoscope. The results show a hit rate of over 89% for predicting the movement of the surgeon’s instruments, leading to 29.2% less camera movements and improved visibility.^27,32^

### Knot-Tying

Although in open surgery, knot-tying is part of basic skills and a relative fast procedure, in minimally-invasive surgery, laparoscopic knot-tying can take up to three minutes for a single knot to complete. Mayer et al.^32^ described a system to speed up the knot-tying based on RNNs in robotic heart surgery. The surgeon presents a sequence (eg, examples of human-performed knot-tying) to the network and, an RNN with long-term storage learns the task. The preprogrammed controller was able to construct a knot in 33.7 seconds, whereas the use of an RNN provided—after learning from 50 previous runs—a speed improvement of almost 25%, producing a knot in 25.8 sec.^27,33^

### Automatic Registration and Tracking of the Bone in Orthopedic Surgery

In computer-assisted orthopedic surgery, registration of the bone plays a vital role as it describes the position of the patient in regard to the surgical system. This way, the surgical site can be correctly aligned according to the preoperative plan. Therefore, the precision of the registration has influence on all the following steps in the procedure. Liu et al.^29^ describe a new way of automatic registration and tracking of the bone, based on depth imaging and deep learning. During surgery, a depth camera repeatedly captures depth images of exposed bone. Using these images, deep neural networks learn to localize, segment, and extract the surface geometry of the target bone. The extracted surface geometry is then compared to a preoperative model of the same bone for registration, making surgical intervention or invasive optical markers superfluous. Ex vivo experiments show a mean translational and rotational error of 2.74 mm and 6.66°, respectively. However, these accuracies are currently lower than conventional intraoperative registration methods based on optical markers.^34,29^

## Discussion

The results of this systematic review study provide an overview of various AI applications currently used for surgical purposes inside the OR. The great majority, of the AI applications have shown superior results in comparison to their non-AI alternatives. However, studies are set up in various pilot settings. The various applications are an indication of multi-field interest in finding use cases for AI in the OR, paired with a need for more clinical research across user settings. Many studies have shown significant technological performance in the field of AI, but only a small minority has been able to situate their impacts and associated changes in current health systems.^35^

According to Rogers’^36^ widely used *Diffusion of Innovations* theory, adoption of innovative technology always involves early and late adopters. During the innovation process, where an individual is motivated to reduce uncertainty about the advantages and disadvantages of an innovation, it is important to emphasize the ethical and legal challenges.^37,38^ Yet, sufficient political, regulatory, organizational, and clinical conditions for AI development and ethical use of sensitive information are still lacking and hence needed to implement AI applications safely and sustainably in the future.^35,39,40^ Additional barriers for the widespread implementation of AI in health care may be unawareness on the topic or solutions, lack of user or implementation knowledge by the medical professionals and their workplace supporters, unresolved questions about ethics or privacy from management, or an insufficient IT infrastructure. Most likely, it will be a combination of these barriers.^41^

While AI, and ML in particular, is receiving more attention in surgery, it is obviously not the only field of medicine in which the use of AI is growing.^27^ The surgical field may be able to learn from the use of AI in other medical fields. For example, in oncology, research has demonstrated that ML applications can be of great help for the diagnosis or detection of cancer.^42-44^ In cardiology, AI techniques are capable of reading electrocardiograms, and by integration with electronic medical records of patients, heart failure can be detected early on with reduced mortality as outcome.^45–47^ In anesthesiology, ANNs are used to monitor the depth of anesthesia, and ML techniques are able to predict hypotension during surgery.^48,49^ And now, during the current COVID-19 pandemic, more AI applications and studies have been initiated.^19,50,51^ The Guangdong Second Provincial General Hospital, for example, plans to incorporate AI image recognition into their infection control system to provide real-time monitoring and an aid for minimizing the risk nosocomial COVID-19 infection. The observing system aims to enhance the sensitivity and accuracy of instant detection in negative pressure isolation wards, which offers creative assistance to combat the COVID-19 outbreak.^50^ This application may also be used in the OR to minimize the risk of surgical infection.

Indeed, AI in health care has presented some promising and impressive results and is a fertile area of research, as Challen et al.^52^ concluded in their review. However, as this study shows the multilingual character of AI in surgery, AI is a complicated and comprehensive field of study. The rapid pace of change, diversity of different techniques, and multiplicity of tuning parameters make it difficult to get a clear picture of how accurate these systems might be in clinical practice or how reproducible they are in different environments.^52^ A realistic perspective is needed, balancing the potential for improvement against the risk of negative outcomes. As Yu et al.^8^ also concluded, we need to acknowledge the brittleness of these systems, the importance of defining the correct frameworks for their application, and ensure rigorous quality control, including human supervision, to unwanted outcomes. Rigorous prospective trials in a diverse patient population and clinical review of atypical feature statistics are needed, to safeguard the value and coherency of the collected data.^8,52^ It is therefore wise to attract knowledge coming from ML experts, ethicists, and lawyers, next to healthcare professionals, to decide on proper fit of use case and safety of AI systems.

This study has some limitations to take into account. First, as this is a review study, unpublished data and gray literature, such as technical reports, are not included, which may have strengthened the results. Moreover, the results may have been influenced by a publication bias, especially, because—as this is study shows—AI assistance in the OR is still in its infancy. Park et al.^53^ acknowledged the problem of irregular research designs in medical AI studies. This is also displayed by the significant variability in the way results are reported, making it very difficult to combine and compare data across studies. This results in the realization that before any AI tool can be used in clinical practice, it requires confirmation of its clinical utility by undergoing thorough research. In their article, they therefore described and reviewed essential methods on the design of such studies, like the importance of using an adequate external dataset, crucial to the clinical evaluation of AI in medicine.^53^

Second, the applications of AI discussed in this study are, although interesting in their pilot effort, not ready for large-scale clinical practice.^54^ AI is not yet able to detect causal relationships in data at a necessary level for clinical implementation to rely on, nor is it able to produce truly automated interpretations of its analyses.^54^ Before these implications can be clinically and safely applied in the OR on a bigger scale, future studies should focus on clinical studies, with data from actual patients.^39^

## Conclusion

AI systems inside the OR, if well-designed, embedded, and researched, may have a promising future in the OR environment. It may support surgical decision-making, improve surgical precision, reduce manpower, improve workflow, increase surgical safety, and some day it may even carry out some autonomous functions.^6–8,16,21^ In the not so distant future, evolving technology like the OR black box, with integrated deep learning algorithms, may prove to be of great help in analyzing and optimizing workflow and outcome in real time.^55^ Indeed, the application and implementation of AI inside the OR still has several challenges to overcome. However, evidence-based research adding to the body of knowledge concerning applications of AI inside the OR is moving quickly. Healthcare professionals ought to accept the fact that we need AI in order to optimize future circumstances in the OR and ultimately, surgical quality and safety.^14,55,56^

## Supplemental Material

sj-pdf-1-sri-10.1177_1553350621996961 – Supplemental Material for A Review on the Current Applications of Artificial Intelligence in the Operating Room Click here for additional data file. Supplemental Material, sj-pdf-1-sri-10.1177_1553350621996961 for A Review on the Current Applications of Artificial Intelligence in the Operating Room by David C. Birkhoff, Anne Sophie H.M. van Dalen and Marlies P. Schijven in Surgical Innovation
