# Supplementary material for: A Review on the Current Applications of Artificial Intelligence in the Operating Room
Source: Surg Innov. 2021 Feb 24;28(5):611–9. doi: 10.1177/1553350621996961 (PMC8450995; doi:10.1177/1553350621996961)
Supplement: sj-pdf-1-sri-10.1177_1553350621996961 – Supplemental Material for A Review on the Current Applications of Artificial Intelligence in the Operating Room [file sj-pdf-1-sri-10.1177_1553350621996961.pdf]

APPENDIX A. Search details 27th of August 2020

Pubmed Database Search Query:

- **2010 to 2020 (n=211):**

(Artificial intelligence[MeSH] OR Computational Intelligence[tiab] OR Machine Intelligence*[tiab] OR Computer Reasoning[tiab] OR AI[tiab] OR Computer Vision System*[tiab])*

AND

(Operating rooms[MeSH] OR Operating room[tiab] OR operating theater*[tiab] OR operating suite*[tiab] OR operation suite*[tiab] OR operating theatre*[tiab])*

AND

(General surgery[MeSH] OR Surgery[tiab] OR operation[tiab] OR surgical procedure*[tiab] OR operative therap*[tiab] OR operative procedure*[tiab] OR peroperative procedure*[tiab] OR perioperative procedure*[tiab] OR preoperative procedure*[tiab] OR intraoperative procedure*[tiab])*

History and Search Details — Download — Delete

Search	Actions	Details	Query	Results	Time
#3	•••	>	Search: **(Artificial intelligence[MeSH] OR Computational Intelligence*[tiab] OR Machine Intelligence*[tiab] OR Computer Reasoning[tiab] OR AI[tiab] OR Computer Vision System*[tiab]) AND (Operating rooms[MeSH] OR Operating room*[tiab] OR operating theater*[tiab] OR operating suite*[tiab] OR operation suite*[tiab] OR operating theatre*[tiab]) AND (General surgery[MeSH] OR Surgery[tiab] OR operation*[tiab] OR surgical procedure*[tiab] OR operative therap*[tiab] OR operative procedure*[tiab] OR peroperative procedure*[tiab] OR perioperative procedure*[tiab] OR preoperative procedure*[tiab] OR intraoperative procedure*[tiab])** Filters: **from 2010 - 2020** Sort by: **Most Recent**	211	11:20:34

Embase Database Search History:

- **2010 to 2020 (n=57):**

(Artificial intelligence OR Computational Intelligence OR Machine Intelligence* OR Computer Reasoning OR AI or Computer Vision System*)*

AND

(Operating room or operating theater* or operating suite*)*

AND

(General surgery OR Surgery or operation OR surgical procedure* OR operative therap* OR peroperative procedure OR perioperative procedure OR preoperative procedure* OR intraoperative procedure*)*

▼ Search History (2) View Saved

# ▲	Searches	Results	Type	Actions	Annotations
1	((Artificial intelligence or Computational Intelligence* or Machine Intelligence* or Computer Reasoning or AI or Computer Vision System*) and (Operating room* or operating theater* or operating suite*) and (General surgery or Surgery or operation* or surgical procedure* or operative therap* or peroperative procedure or perioperative procedure or preoperative procedure* or intraoperative procedure*)).mp. [mp=title, abstract, heading word, drug trade name, original title, device manufacturer, drug manufacturer, device trade name, keyword, floating subheading word, candidate term word]	73	Advanced	Display Results More	
2	limit 1 to yr="2010 -Current"	57	Advanced	Display Results More	

Save Remove Combine with: AND OR

Cochrane Library Search Query:

- ➔ **2010 to 2020 (n=5):**

(Artificial intelligence OR Computational Intelligence OR Machine Intelligence* OR Computer Reasoning OR AI or Computer Vision System*)*

AND

(Operating room or operating theater* or operating suite*)*

AND

(General surgery OR Surgery or operation OR surgical procedure* OR operative therap* OR peroperative procedure OR perioperative procedure OR preoperative procedure* OR intraoperative procedure*)*

5 Cochrane Reviews matching **(Artificial intelligence OR Computational Intelligence* OR Machine Intelligence* OR Computer Reasoning OR AI or Computer Vision System*) AND (Operating room* or operating theater* or operating suite*) AND (General surgery OR Surgery or operation* OR surgical procedure* OR operative therap* OR peroperative procedure OR perioperative procedure OR preoperative procedure* OR intraoperative procedure*) in Title Abstract Keyword - with Cochrane Library publication date Between Jan 2010 and Jul 2020 (Word variations have been searched)**

Did you mean: *orificial* | *artificialise* | *artificiality*

Cochrane Database of Systematic Reviews
Issue 8 of 12, August 2020

IEEE Xplore database Search Query:

- ➔ **2010 to 2020 (n=27):**

(((“All Metadata”:artificial intelligence) AND “All Metadata”:surgery) AND “All Metadata”:operating room)

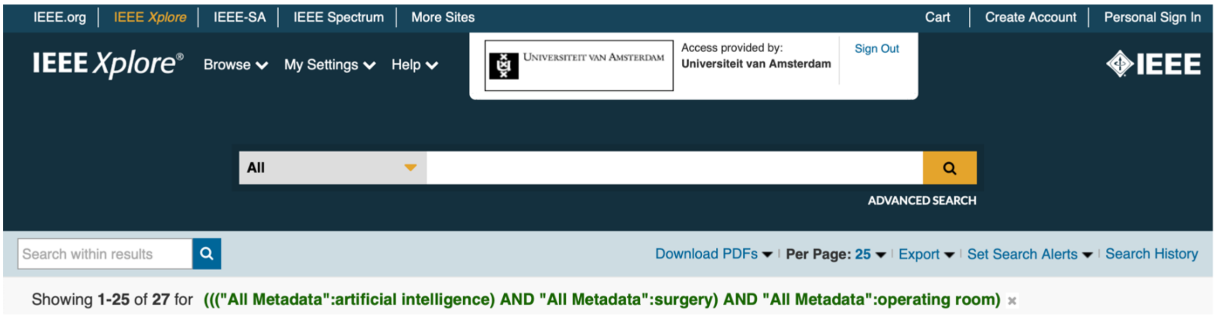

APPENDIX B. Critical appraisal checklist

General checklist for systematic reviews:

➔ Accessible at:

**General systematic review (SR) quality criteria**			
	Yes	No	Can't tell
Does the SR explicitly report and perform a comprehensive and reproducible literature search?			
Does the SR formulate a clearly focused question?			
Does the SR's methods section explicitly state the basis for inclusion or exclusion of primary RCTs?			
Does the SR report data from primary RCTs (e.g., size, interventions used, results from individual RCTs)			
Does the SR assess the methodological quality of primary studies, and take these into account where necessary?			
Meta-analysis: does the SR combine primary studies appropriately?			
Meta-analysis: does the SR state how results are combined statistically?			
Meta-analysis: does the SR report absolute numbers as well as appropriate summary statistics?			
Does the SR discuss the reasons for any variations/heterogeneity between individual RCTs/overall results?			
Does the SR report on the clinical relevance/importance of the results?			
